# Comparing Interfacial Trp, Interfacial His and pH Dependence for the Anchoring of Tilted Transmembrane Helical Peptides

**DOI:** 10.3390/biom10020273

**Published:** 2020-02-11

**Authors:** Fahmida Afrose, Roger E. Koeppe II

**Affiliations:** Department of Chemistry and Biochemistry, University of Arkansas, Fayetteville, AR 72701, USA; fafrose@uark.edu

**Keywords:** transmembrane helix, interfacial aromatic ring, solid-state deuterium NMR, tryptophan, histidine

## Abstract

Charged and aromatic amino acid residues, being enriched toward the terminals of membrane-spanning helices in membrane proteins, help to stabilize particular transmembrane orientations. Among them, histidine is aromatic and can be positively charge at low pH. To enable investigations of the underlying protein-lipid interactions, we have examined the effects of single or pairs of interfacial histidine residues using the constructive low-dynamic GWALP23 (acetyl-GG^2^ALW^5^LALALALALALALW^19^LAG^22^A-amide) peptide framework by incorporating individual or paired histidines at locations 2, 5, 19 or 22. Analysis of helix orientation by means of solid-state ^2^H NMR spectra of labeled alanine residues reveals marked differences with H^2,22^ compared to W^2,22^. Nevertheless, the properties of membrane-spanning H^2,22^WALP23 helices show little pH dependence and are similar to those having Gly, Arg or Lys at positions 2 and 22. The presence of H5 or H19 influences the helix rotational preference but not the tilt magnitude. H5 affects the helical integrity, as residue 7 unwinds from the core helix; yet once again the helix orientation and dynamic properties show little sensitivity to pH. The overall results reveal that the detailed properties of transmembrane helices depend upon the precise locations of interfacial histidine residues.

## 1. Introduction

Membrane-spanning proteins have distinct segments composed of hydrophobic amino acid residues flanked by interfacial loops that tend to be rich in aromatic amino acids. In integral membrane proteins and peptides, it has been found from statistical analysis [1,2,3,4,5,6] and genomic databases [2,7,8] that the aromatic amino acids are not uniformly distributed but rather are significantly localized at the membrane-water interfaces. A major driving force causing the aromatic amino acids to prefer the interfacial location is thought to be the hydrophobic effect, but their rigid ring structure and involvement in hydrogen bonding with lipid carbonyl groups or water molecules, as well as the dipole-dipole interactions in the interface region, are additional contributing factors [9,10,11].

Because membrane proteins are inherently complex in structure, there are difficulties for the structural and functional characterization of membrane proteins using experimental techniques. Implementation of synthetic model peptides has proven invaluable to overcome some of the difficulties of research focused on fundamental principles of protein-lipid interactions. A good example model peptide is GWALP23, acetyl-GGALW^5^LALALALALALALW^19^LAGA-amide [12,13], which not only minimizes issues related to the complexity of biological membrane proteins but also allows for a wealth of information derived from single or multiple residue replacements throughout its sequence. In historical sequence, the GWALP23 family peptides were derived from “WALP” model peptides [14] by reducing the number of tryptophan (W) residues [13]. Since its development, GWALP23 has been used as the “host” for several “guest” amino acid residues within its sequence, to study the influence of particular amino acid side chains, such as those having aromatic, charged or polar residues in various locations on the scaffold of a well-defined transmembrane helix.

GWALP23 was developed by introducing two interfacial glycine residues, G2 and G22, in place of tryptophans [13]. These paired W to G substitutions lead to less molecular dynamic averaging than was observed with the WALP23 [15] or WALP19 [16] helices [13,17]. Indeed, when four tryptophans flank a core helix, whether at positions 2, 3, 21, 22 or at positions 2, 5, 19, and 22, the high dynamic behavior of the transmembrane peptide helix is maintained [17,18], suggesting potential “competition” of aromatic rings for stable interactions with lipid head groups at a membrane interface [19]. In this context, a choice of histidine for these positions allows one to verify this scenario and to compare the effect of imidazole versus indole rings for the dynamics of a transmembrane peptide helix. 

With the ability to provide not only an aromatic ring but also interaction between a positively charged imidazole side chain and negatively charged lipid head groups, histidine offers the potential to promote protein-lipid interaction at the interface in membrane proteins [20,21,22,23]. Yet this residue is not as frequently present as tyrosine or tryptophan at the membrane-water interface [10,24,25,26]. Another recent study tested this hypothesis by mutating the highly conserved F161 residue of the eight-stranded OMP enzyme PagP to H161 and generating a library of PagPX160H mutants with X160 being any amino acid. The analysis resulted in a thermodynamically unstable PagP β-barrel because of the absence of a compact structure [27]. The stabilization was then recovered partially by incorporating a hydrophobic residue (Y, M, I, V, L) at the X position when H161 was present. We seek to gain additional information about the impact of interfacial His residues for the biophysical properties of transmembrane helices.

For the experiments described below, we have incorporated combinations of histidine residues H2, H22, H5 and H19, for comparison with properties conferred by G2, W2, W5, W19, G22 and W22; to address issues of transmembrane helix orientation, stability and dynamics when individual or pairs of interfacial side chains are changed. Initially, histidine residues were incorporated at positions 2 and 22, outside the tryptophan cage of GWALP23, to give the sequence acetyl-GH^2^ALW^5^LALALALALALALW^19^LAH^22^A-amide (Table 1). Later, for comparison, one histidine was omitted from either position 2 or 22 and replaced with glycine, to yield peptides anchored by two aromatic chains (imidazole and indole) at one end, while the other side is anchored only by Trp. These substitutions resulted in two asymmetrically anchored peptides, H^2^GWALP23 and H^22^GWALP23. Further investigations involved replacement of W5 or W19 with H5 or H19 which resulted in two more GWALP23 mutants with a single histidine at the N- or C-terminal end and tryptophan at the other end. In addition, a histidine pair was introduced to replace both W5 and W19, to examine the properties displayed by a transmembrane helix flanked only by two histidine residues, without tryptophan, and to compare the similarities and/or differences of having histidine instead of tryptophan or tyrosine, which was previously studied [28](Table 1).

**Figure 1:**
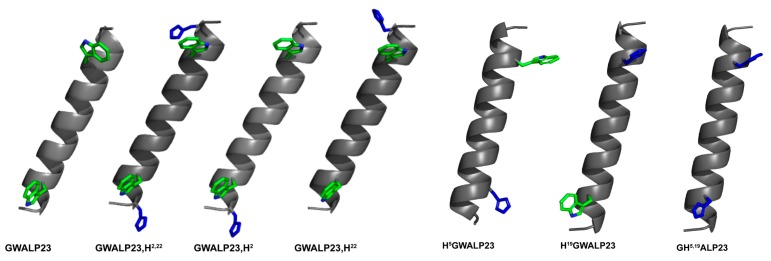
Pymol [29] representation of GWALP23 family peptides with histidine mutations in different locations. The His imidazole rings are blue, and the Trp indole rings are green with blue nitrogens. The depicted side-chain orientations and helix tilt are arbitrary. The sequences are shown in Table 1.

Some of the approximate locations for the histidines in the respective helices are illustrated in Figure 1. The results will reveal that H2 and H22 confer quite similar properties as G2 and G22, but very different from W2 and W22. At the slightly more interior positions 5 and 19, residues H5 and H19 confer similar properties to W5 and W19, as well as Y5 and Y19. We investigate also the pH dependence of helix properties, or lack thereof, and partial unwinding of the core transmembrane helix when particular histidines are present. Histidine 5, indeed, may disrupt the core helix under some conditions. While the His residues may titrate, the core helix often shows little response to the titration of His residues near the membrane interface. This minimal response to pH contrasts with the larger response of similar transmembrane helices to the titration of more buried His residues.

## 2. Materials and Methods 

### 2.1. Synthesis and Purification of ^2^H-labeled Peptides

N-Fmoc protected amino acids and rink amide resins were purchased from NovaBiochem (San Diego, CA) and (Louisville, KY). Commercial L-alanine, deuterated at Cα and Cβ carbons (Ala-d_4_), from Cambridge Isotope Laboratories (Andover, MA) was derivatized with an Fmoc protecting group as described previously [30,31]. ^1^H-NMR spectroscopy was used to confirm the successful Fmoc-Ala-d_4_ synthesis. DLPC and DOPC synthetic lipids were purchased from Avanti Polar Lipids (Alabaster, AL). Histidine and tryptophan side chains were protected with trityl and t-butoxycarbonyl protecting groups and were purchased from Novabiochem and Bachem.

Peptides were synthesized on a model 433A solid-phase peptide synthesizer (Applied Biosystems; Foster City, CA, USA) using modified FastMoc® chemistry, with extended deprotection and coupling times where needed. Two deuterium-labeled alanines at ~50% and 100% isotope abundance levels were incorporated in a single peptide, allowing the NMR signals to be distinguished and assigned based upon the relative intensities. The final residue on each peptide was acetyl-Gly to yield a blocked, neutral N-terminal.

After synthesis, peptides were simultaneously deprotected and cleaved from rink amide resin with a peptide cleavage solution containing TFA:phenol:triisopropylsilane:water in a 85:5:5:5 ratio. Crude peptides were purified on a reversed-phase HPLC octyl-silica Zorbax Rx-C8 column with 9.4 × 250 mm dimensions and 5 μm particle size from Agilent Technologies (Santa Clara, CA, USA). A gradient of 92–96% (for the H^2^, H^22^ and H^2,22^ peptides), 94–98% (for the H^5^ and H^19^ peptides) or 88–92% (for H^5,19^ peptide) methanol (with 0.1% trifluoroacetic acid (*v*/*v*)) at a flow rate of 1.7 mL/min or about 15 min was used for each peptide purification. Finally the peptides were lyophilized multiple times with 1:1 mixture of acetonitrile:water to ensure complete removal of TFA. Peptides without Trp (e.g., H^5,19^GWALP23) were detected based on absorbance at 220 nm, whereas the Trp-containing peptides were detected based on absorbance at 280 nm, using a molar extinction coefficient of 5600 M^−1^cm^−1^ for each Trp in the sequence [31]. The identity of peptide and deuteration pattern were confirmed by MALDI mass-spectrometry (Figures S1 and S2).

### 2.2. Circular Dichroism (CD) Spectroscopy

Circular dichroism measurements were performed on peptides incorporated into small unilamellar vesicles of DLPC or DOPC at 1/60 (mol/mol) peptide/lipid (P/L) in unbuffered water, obtained by ultrasonic treatment. Spectra were recorded at 22 °C on a Jasco J-1500 spectropolarimeter, using a scan speed of 20 nm/min in a cell of 1.0 mm path length, 1.0 nm band width, and 0.1 nm slit. An average of six scans was recorded to enhance the signal-to-noise ratio.

### 2.3. ^2^H and ^31^P NMR Spectroscopy Using Oriented Bilayer Samples

Solid-state NMR samples were prepared, as described previously [16] using macroscopically aligned lipid bilayers. After quantification of obtained purified peptides, 1.33 μmol (~ 3 mg) peptide was aliquoted and mixed with 80 μmol of lipid to achieve 1:60 P/L molar ratio. Solvents were removed under a stream of nitrogen and samples were dried under vacuum. The mixture of peptide-lipid was redissolved in methanol:water 95:5 and distributed evenly among 34 glass slides (0.08 × 4.8 × 23 mm; Marienfeld, Germany) and then dried under vacuum for 48 h. The dried P/L films were then hydrated with buffers of required pH, made from ^2^H-depleted water (Cambridge), sealed in a glass cuvette and incubated at 40 °C for minimum 24 h (or more depending on pH) before measurement.

Solid-state NMR spectra were recorded at 50 °C using two Bruker Avance spectrometers (Billerica, MA), each operating at a proton frequency of 300 MHz. For each NMR sample spectra were recorded with the membrane normal either parallel (β = 0°) or perpendicular (β = 90°) to the applied magnetic field. At first the samples were subjected to ^31^P NMR, decoupled with ^1^H, to confirm bilayer alignment within each sample. For ^2^H NMR spectroscopy, spectra were obtained using a quadrupole echo pulse sequence [32], with full phase cycling, 3.2 μs pulse length, 105 μs echo delay and 120 ms recycle delay. Typically, 0.9 to 1.5 million free induction decays were recorded, with applied exponential weighting function of 100 Hz line broadening (prior to Fourier transformation). For a peptide with fast averaging around the lipid bilayer normal the ^2^H quadrupolar splittings (Δν_q_) observed at β = 90° have absolute magnitude of ½ those at β = 0° [16,33]. 

### 2.4. Analysis of Helix Orientation and Dynamics from ^2^H NMR Data

The ^2^H NMR signals from C_β_D_3_ groups of Ala-d_4_ residues of peptides were analyzed according to the Geometric Analysis of Labeled Alanines (GALA) method described previously [15,16]. This is a semi-static analysis based on a relationship between the alanine C_β_D_3_ quadrupolar splitting (Δν_q_) and the angle θ between the alanine C_α_-C_β_ bond vector and the applied magnetic field [16]. When fitted with at least four and preferably six or more data points around a helical wheel [15,16], GALA analysis provides a 360° quadrupolar wave plot which reveals the helix orientation with respect to a vector normal to the lipid-bilayer membrane, by fitting and reporting the average tilt τ of the helix axis, the azimuthal rotation ρ, and a principal order parameter S_zz_ as variables. Additionally, we have employed another method called “Modified Gaussian Analysis” [18,19] to describe in more detail the helix dynamics and to reconfirm and elaborate the results from the GALA analysis.

## 3. Results

In this study, we investigate the interactions of interfacial histidines, present alongside tryptophans, flanking a transmembrane segment that is similar to helices of biological proteins. 

The peptide molecular masses and deuteration patterns were verified by MALDI-TOF mass spectrometry (Figures S1 and S2 of the Supporting Information). Because all of the peptides have a hydrophobic core composed of alternating Leu-Ala residues, they are expected to adopt an α-helical conformation. The secondary structures were confirmed by CD spectra for the H^2,22^, H^2^, H^22^, H^5^, H^19^ and H^5,19^ peptides incorporated into hydrated DLPC and DOPC bilayer membranes. Each peptide exhibited a mean residue ellipticity profile typical of an α-helix, characterized by minima at 208 and 222 nm and a ratio ε_222_/ ε_208_ between 0.74 and 0.86 (Figures S3 and S4 of the Supporting Information). For aligned lipid/peptide samples, the lipid alignment was checked by means of solid-state ^31^P NMR (Figures S5 and S6). The characteristic ^31^P resonances for the β=0° and β=90° bilayer orientations confirm very good alignment of the lipid membranes of DLPC or DOPC (Figures S5 and S6) when various peptide helices are present. 

### 3.1. Comparison of H with G, K, R and W at Positions 2 and 22

**Figure 2:**
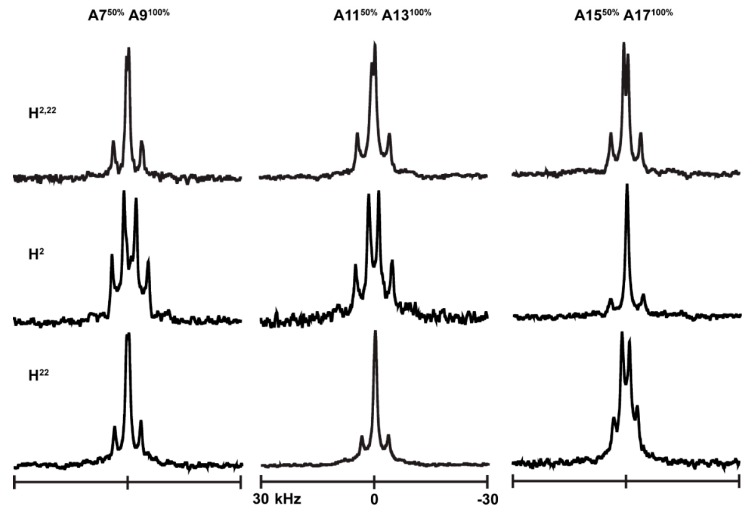
^2^H NMR spectra of H^2,22^WALP23, H^2^GWALP23 and H^22^GWALP23in DOPC lipid bilayers. The samples were hydrated with acetate buffers of pH 4. Temperature 50 °C; β =90° sample orientation.

Solid-state ^2^H NMR spectra reveal well-defined signals for the ^2^H-Ala methyl groups of H^2,22^WALP23 in DLPC and DOPC lipid bilayers (Figure 2 top panel). Each ^2^H-label at the core of this peptide also produces a distinct quadrupolar splitting magnitude (|Δν_q_|). These well-resolved signals as well as the quadrupolar splitting values are indicative of a single predominant tilted transmembrane orientation in each lipid bilayer. The spectra observed previously for X^2,22^WALP23 peptides with similar mutations reveal quite similar feature when X = G, R or K but quite different features when X = W [13]. 

To investigate how histidine side chains in the same positions affect the peptide helix behavior, we have compared the spectra and ^2^H quadrupolar splitting magnitudes of the H^2,22^ peptide with the G^2,22^; K^2,22^; R^2,22^ and W^2,22^ analogs. The measured quadrupolar splittings (Table 2) for the H^2,22^ analog are quite different from those for the W^2,22^ peptide. H^2,22^WALP23 displays a wide |Δν_q_| range from 4–35 kHz in DLPC and 1–17 kHz in DOPC (Table 2). Conversely, these ranges for W^2,22^WALP23 are moderate in DOPC (about 2–14 kHz) and significantly narrow in DLPC (1–14 kHz). Interestingly, the Δν_q_ magnitudes of H^2,22^WALP are in close proximity to those for G^2,22^WALP, R^2,22^WALP or K^2,22^WALP (Table 2 and [13]), which implies that the orientations of this double-histidine peptide in DLPC and DOPC lipid membranes are likely to be similar to those of the G^2,22^WALP, R^2,22^WALP and K^2,22^WALP helices. 

**Figure 3:**
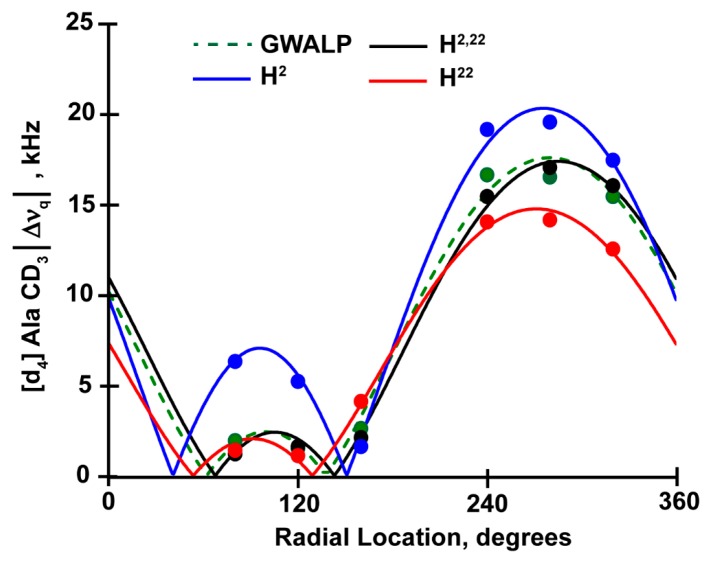
Quadrupolar wave plot for oriented GWALP23 family peptides with histidine residues H2 and/or H22 in DOPC lipid bilayers.

**Figure 4:**
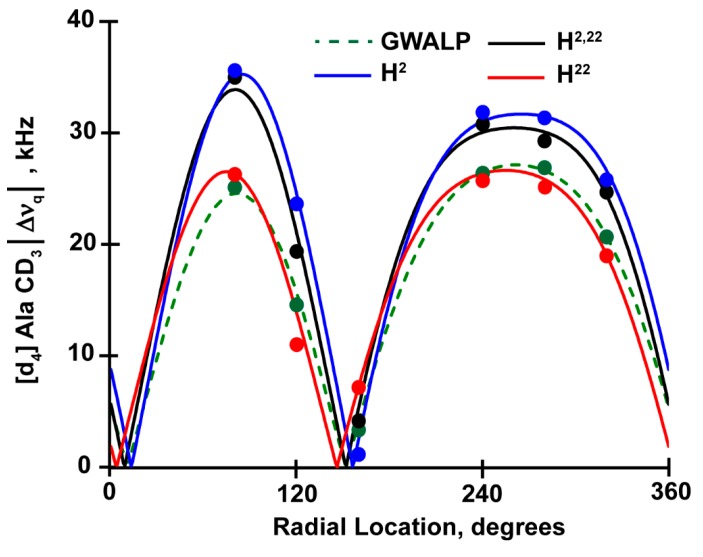
Quadrupolar wave plot for oriented GWALP23 family peptides with histidine residues H2 and/or H22 in DLPC lipid bilayers.

The Geometric Analysis of Labeled Alanines (GALA) method reveals only minor changes in the orientations of peptide helices when G2 and G22 are replaced by histidines (Figure 3 and Figure 4, Table 3). The H^2,22^WALP23 peptide exhibits a tilted transmembrane orientation that scales with the membrane thickness to adjust hydrophobic mismatch. In DLPC, the apparent tilt (τ) angle of H^2,22^WALP23 is near 26^o^, about 5^o^ higher than for G^2,22^WALP23. This minor change in tilt angle is absent when the helix is moved to thicker DOPC membrane, meaning the tilt angles of H^2,22^WALP23 and G^2,22^WALP23 are essentially identical (about 6^o^) in DOPC. As expected, the modified Gaussian analysis [19] more accurately addresses the dynamic averaging and predicts slightly higher tilt angles, about 10^o^ for the G^2,22^ and H^2,22^ helices in DOPC (Table 3). The observed azimuthal rotations (ρ) for H2 and H22, on the other hand, are slightly different in DOPC and almost same in DLPC. These observations remain consistent when one compares the results of H^2,22^WALP23 with R^2,22^WALP23 and K^2,22^WALP23 (Table 3).

### 3.2. Comparison of H^2,22^ with H^2^ and H^22^

For further analyzing the residues that control the orientation of the H^2,22^WALP23 helix in lipid bilayers of DLPC and DOPC, we changed one histidine to glycine, resulting in H^2^GWALP23 and H^22^GWALP23 (Table 1). Figure 2 displays and compares the ^2^H NMR spectra for labeled core alanine residues 7, 9, 11, 13, 15 and 17 for H^2,22^WALP, H^2^GWALP and H^22^GWALP peptides in DOPC lipid bilayers. The spectra for the same peptides in DLPC lipid membranes are shown in Figure S7. The single-histidine H^2^ and H^22^ peptides exhibit sharp signals and well-resolved spectra for labeled core alanines in both lipids, with wide ranges of ^2^H quadrupolar splittings (Table 2). These ranges indicate a well-defined tilted orientation for each helix, with low to moderate dynamic averaging [17,19].

Comparing the quadrupolar wave plots and the apparent tilt (τ) and azimuthal rotation (ρ), obtained from GALA analysis (Figure 3 and Figure 4, Table 3), it is evident that all three α-helices with a histidine residue close to one or both termini adopt stable transmembrane orientations in bilayers of two different thicknesses (C12:0 and C18:1), with little change in the overall orientation. To adjust the hydrophobic mismatch between lipid and peptide, all three peptides scale their tilt (τ) and rotation angles (ρ) when moved from thinner DLPC to thicker DOPC membranes. For the H^2^ peptide in DLPC bilayers the resulting tilt angle is identical to the observed tilt for H^2,22^ peptide (Table 3), whereas in DOPC it is ~3^o^ more tilted. The H^22^ peptide, conversely, has same tilt magnitude as H^2,22^ in DOPC but ~3^o^ less in DLPC. The observed azimuthal rotation angle varies by no more than 10° among the three helices in the two lipid membranes. The combined results suggest that the presence of histidine, at the N- or C-terminal or both, does not much affect the overall helix orientation. These features suggest that the more interior Trp residues at positions 5 and 19 remain the primary determinants of the helix tilt and rotation.

Results from a modified-Gaussian analysis of the helix dynamics [18,19] generally agree with the predictions of the GALA calculations. The helix with H2 displays only modest values of rotational slippage (σρ) ranging from about 36° in DLPC to 46° in DOPC (Table 3). The H22 isomer, on the other hand, has a very low σρ value of 8° in DLPC but a significantly higher value of about 70° in DOPC (Table 3). When the two histidines are combined in a single peptide to produce the H^2,22^WALP helix, the rotational slippages are intermediate, with values of about 16° in DLPC and 56° in DOPC (Table 3). These results indicate modest motional differences, although the average tilt and rotation of the H^2^ and H^22^ helix isomers are not very different. 

### 3.3. Comparison of H^5,19^ with W^5,19^ and mixed Interfacial Pairs, H^5^W^19^ and W^5^H^19^

Considering residue H5, when comparing the individual Δν_q_ values of all core alanines of H^5^GWALP23 with those for the -W^5^, -Y^5^ and -F^5^ analogs, one notices that these magnitudes are within 1–5 kHz in each case (Figure S8, Table 2). These results suggest relatively similar membrane orientations and dynamic properties for the -H^5^, -Y^5^, -F^5^ and -W^5^ GWALP23 model peptides, with possibly a slight increase in the helix tilt when histidine is present at the N-terminal membrane-water interface. 

In the case of C-terminal replacement of Trp to His, the range of quadrupolar splittings for H^19^GWALP23 (4–21 kHz) is slightly smaller compared to the N-terminal histidine mutant H^5^GWALP23 (Table 2), but are relatively consistent with those seen previously for GWALP23 with W^19^ or the Y^19^ substitution (0.5–15 kHz), implying that these GWALP23-like peptides are tilted to similar extents and exhibit similar dynamics in DOPC lipid bilayer membranes. Moving to the double mutation where W5 and W19 are substituted by H5 and H19, the ^2^H quadrupolar splittings for the six labeled alanines of the Leu-Ala core of GH^5,19^ALP indeed are comparable to those of H^5^ and H^19^GWALP23. Thus, the results for single and double histidine exchanges at positions 5 and 19 are consistent when compared with similar Tyr and Trp analogs.

**Figure 5:**
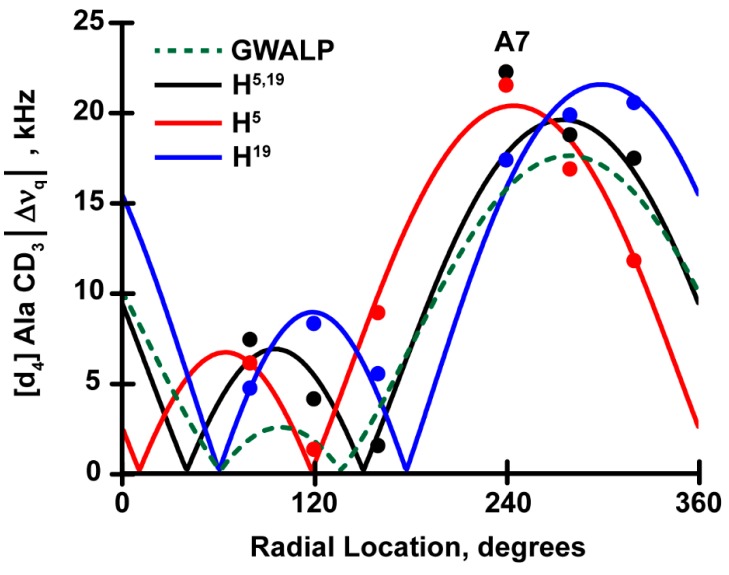
Quadrupolar wave analysis for oriented H^5^GWALP (red), H^19^GWALP (blue) and GH^5,19^ALP23 (black) peptides in DOPC lipid bilayers. The analysis for the host peptide GW^5,19^ALP23 shown as a dotted green wave.

To analyze and verify the helix orientations and motional averaging, we used the “Geometric Analysis of Labeled Alanines (GALA)” method [16]. The results from this analysis, listed in Table 3 and illustrated in Figure 5, demonstrate that the hydrophobic α-helices anchored by one histidine and one tryptophan as well as two histidines H5 and H19 with no tryptophan adopt very similar transmembrane orientations. The resulting tilt angles for these new peptides in DOPC are in the proximity of 9^o^, which is slightly larger than observed for the parent W^5,19^ peptide (Table 3). Indeed the results are comparable to those with various combinations of Trp and/or Tyr at positions 5 and 19 [28]. These comparisons suggest that the presence of H5 or H19 or both confers similar helix properties as for other aromatic residues tyrosine or tryptophan in the same positions.

With minor change in tilt angles, the Trp to His mutations confer some changes in azimuthal rotation (ρ) about the helix axis. The H5 and H19 peptides show approximately 30^o^ and 20^o^ changes in rotation in DOPC lipid membrane compared to the W5,19 counterpart, but the direction of rotational change is opposite for the H5 and H19 substitutions (Table 3). When both H5 and H19 are present, the changes appear to cancel each other and thereby the preferred rotation of double-histidine helix does not differ much from that of the parent GWALP23 helix. 

For each peptide under consideration here, the modified Gaussian approach for estimating the helix orientation and dynamics, [18,19] shows overall agreement with the semi-static GALA analysis, with similar predictions for the helix tilt (τ_0_) and rotation (ρ_0_), and low estimates for the rotational slippage (σρ) values, not exceeding 40°. (Table 3). 

A noteworthy feature observed here is that the ^2^H |Δν_q_| magnitude for deuterated alanine A7, near the beginning of the core helix, fails to fit the core helix backbone geometry when H5 is present. Regardless of whether W19 or H19 is present, the helix with H5 is markedly unraveled from the transmembrane core helix in bilayers of DOPC (Figure 5). This type of unwinding from the N-terminal of the core helix has been observed previously for H^4,5^GWALP23, when two adjacent histidine residues are present N-terminal to the core helix [34]. Now the extended unraveling can be ascribed to residue H5. Because some fraying of both ends has been observed as a general feature for several GWALP23-like model peptides and is thought to have a significant contribution to the dynamic stability of transmembrane peptides [34,35,36], it is likely that the extended unwinding caused by H5 and involving residue A7 may play an important role for the helix orientation, dynamics and stability. Interestingly, by contrast, residue H19 does not confer additional helix fraying of the C-terminal of the core transmembrane helix. 

### 3.4. Ionization of Histidines

**Figure 6:**
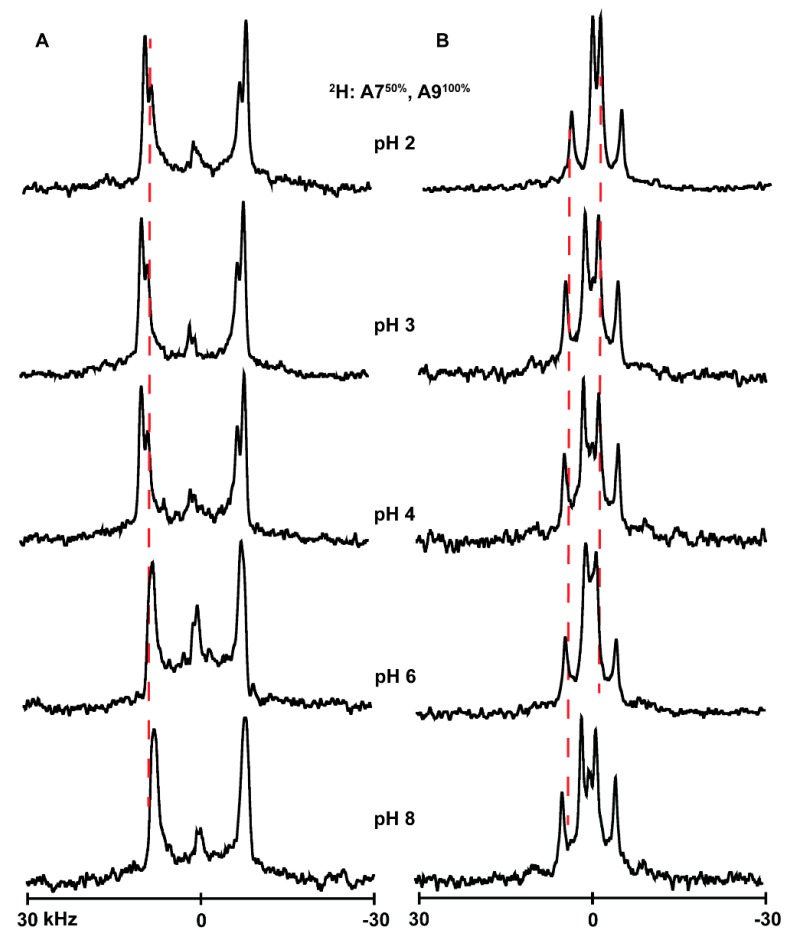
Selected ^2^H NMR spectra to show the pH dependence of resonances for labeled A7 and A9 of H^2,22^WALP23 in DLPC (**A**) and DOPC (**B**) lipid bilayers. Temperature 50 °C; β = 90° sample orientation.

**Figure 7:**
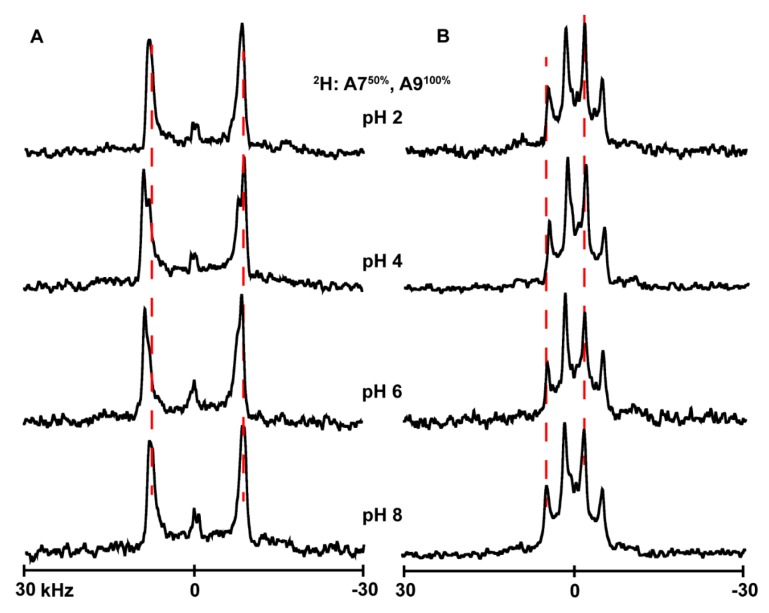
Selected ^2^H NMR spectra to show the pH dependence of resonances for labeled A7 and A9 of H^2^GWALP23 in DLPC (**A**) and DOPC (**B**) lipid bilayers. Temperature 50 °C; β = 90° sample orientation.

**Figure 8:**
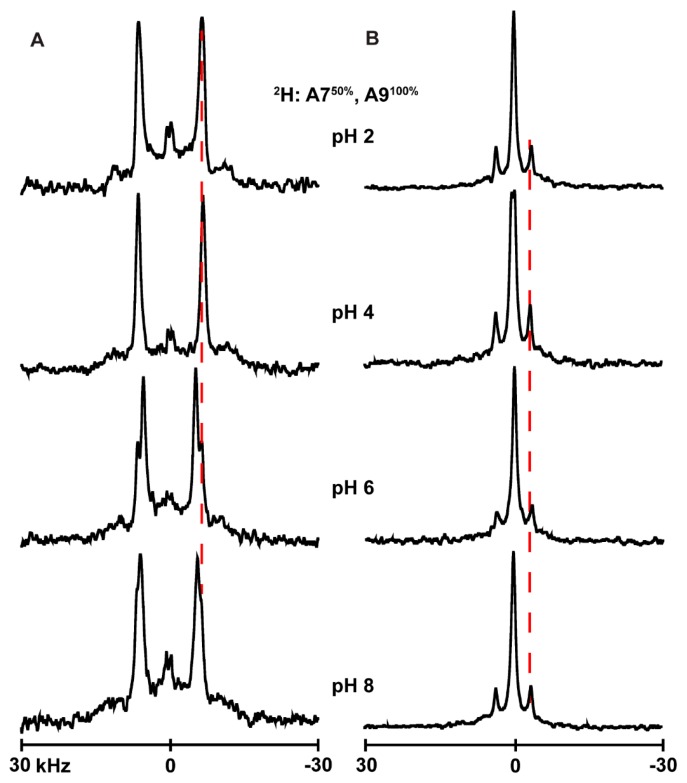
Selected ^2^H NMR spectra to show the pH dependence of resonances for labeled A7 and A9 of H^22^GWALP23 in DLPC (**A**) and DOPC (**B**) lipid bilayers. Temperature 50 °C; β = 90° sample orientation.

In attempts to determine the titration point of the histidine side chain when present at different locations of the transmembrane α-helices, we used solid-state NMR to monitor the changes in the quadrupolar splittings of ^2^H-labeled alanine residues A7 and A9 of several of the peptides studied here. The underlying concept is that a change in ionization might affect the properties of the global helix which in turn might alter aspects of the ^2^H NMR spectra [37]. The spectral changes with pH are nevertheless small to nonexistent for many of these particular helices with interfacial His residues. Indeed, changing the pH environment of peptides from acidic to basic (pH 2–8) causes little change in the spectral quality or the magnitudes of the quadrupolar splittings (|Δν_q_|) for labeled alanines of H^2,22^WALP23 (Figure 6B), H^2^GWALP23 (Figure 7B) or H^22^GWALP23 (Figure 8B) in DOPC bilayers. For the DLPC membranes, there are some minor changes in the signals between pH 4 and pH 6, with the A7 signal being more visible and slightly shifted from the A9 signal (Figure 6A, Figure 7A and Figure 8A). Nevertheless, these changes are not sufficient for plotting reliable titration curves. Therefore, it is probable histidines H2 or H22 titrate with rather normal pK_a_ values, similar to the aqueous value, but that the transmembrane helices do not respond to such titration.

**Figure 9:**
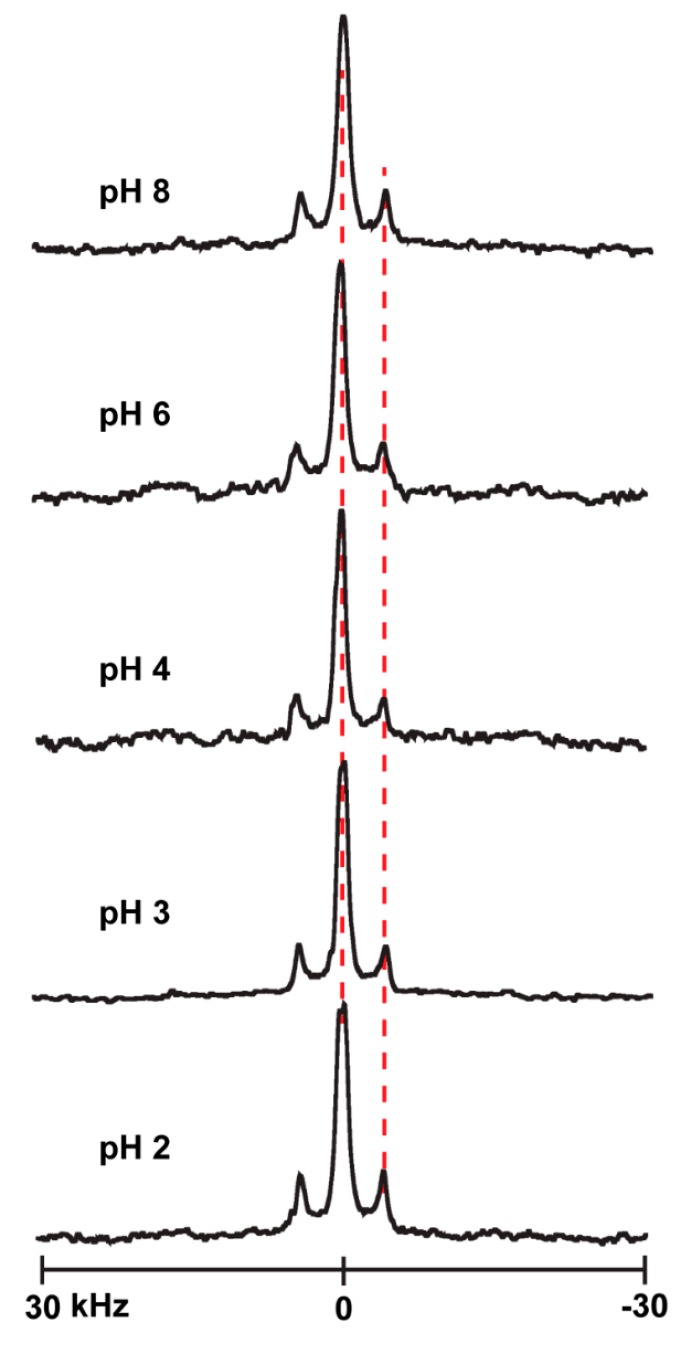
Selected ^2^H NMR spectra to show the pH dependence of resonances for labeled A7 and A9 of GH^5,19^ALP23 in DOPC lipid bilayers. Temperature 50 °C; β = 90° sample orientation.

The pH dependence was investigated also for histidines H5 and H19, which are still interfacial yet one helical turn “inside” of the more outer histidines H2 and H22. Likewise when H5 and H19 are present, the ^2^H NMR spectra show relatively little change over a pH range of 2–8 for the helices in DOPC (Figure 9). Similarly, when a single histidine H5 or H19 is present, the spectra also show little response to changes in the pH (Figures S9 and S10). The results indicate that each peptide helix exhibits sharp ^2^H signals for the labeled alanines over this pH range, meaning they adopt well-defined transmembrane orientations, but there is little observable change in the spectra for the H^5^, H^19^ or H^5,19^ helices when the pH is lowered from 8 to 2. Once again, the transmembrane helices do not respond to the titration of interfacial histidine residues present at the terminus of the helix.

## 4. Discussion

To address further the influence of interfacial His residues, we have employed the well-established GWALP23 host peptide framework. The aim has been to the compare the anchoring properties of histidine when present at or near one or both interfaces of a transmembrane helix. A similar comparison was accomplished previously [28,38] where the aromatic residues Tyr and Trp were found to behave quite similarly in each position considered. Here we have further analyzed the system by comparing histidine and its titration behavior with the non-titrating tryptophan as well as tyrosine at the N- and/or C-terminal interfaces of the transmembrane α-helical segment. 

The investigations reveal that neither histidine nor tyrosine alters fundamentally the behavior of GWALP-like peptides. The reports from the ^2^H quadrupolar splittings of central Ala residues in GWALP23 with single or a pair of interfacial histidines do not vary much, whether at positions 5 and 19, or 2 and 22 (discussed later). The resulting ^2^H quadrupolar wave plots observed for the core helices of H^5^GWALP23, H^19^GWALP23 and GH^5,19^ALP23 are similar to those of the parent helix GWALP23. The helix of GWALP23 is moderately dynamic with a tilted transmembrane orientation that changes with the lipid bilayer thickness [13]. With H5 and W19 the new helix adopts a similar well-defined orientation in DOPC, with a 3° higher tilt (τ_o_) and 30° change in azimuthal rotation. The rotational slippage remains modest at about 35°, meaning that the replacement of indole ring side chain with an imidazole one does not affect the helix dynamics. The only important difference for H^5^GWALP23 is a longer unwound N-terminal which not only unravels a portion of the core helix but also includes the mutated H5 residue. When the histidine is moved from position 5 to 19 (with Trp occupying position 5), one observes almost no change in helix tilt but about 50° change in rotation compared to its H5 isomer. The dynamic properties, by means of σρ value, also remain lower and unchanged. But very interestingly, with H19 no additional unwinding or helix fraying is observed. The H^19^GWALP23 peptide stays helical from at least residue 7 to 17, as with GWALP23 but unlike H^5^GWALP23. Furthermore, when both H5 and H19 are present, the fit for σρ does not increase; the helix displays an identical tilted orientation to that of GWALP23 with only moderate rotational slippage and helical wobbling. Indeed, the rotational changes induced by H5 or H19 individually cancel each other when both histidines are present. Notably, nevertheless, for this GH^5,19^ALP23 helix, residue A7 once again falls off the helical wave plot, with a deviation of about 5.0 kHz, resulting in extended N-terminal unraveling, ostensibly from residue 1 to 7, which is also observed for H^5^GWALP23. Thus, one observes that the N-(juxta)-terminal histidine H5 disrupts the core helix integrity but the C-(juxta)-terminal histidine H19 does not. 

With respect to the outer locations, when the Gly residues from positions 2 and 22 of GWALP23 are substituted by positively charged amino acids such as lysine or arginine, the properties of the transmembrane helix remain quite similar, with a small increase in apparent tilt angle and no change in the helix azimuthal rotation (tilt direction) [13]. But when two Trp residues are incorporated in these positions, giving rise to “extra” Trp residues, the direction of tilt becomes less well defined due to rotational slippage regardless of the lipid bilayer thickness [13]. These behaviors are also observed for few other related peptides such as WALP19 [16] and WALP23 [15]. Notably, the common feature among these high dynamic peptides is the presence of multiple tryptophan residues at both ends of the transmembrane segment, which possibly compete with each other for preferred positions and thereby give rise to extensive dynamic averaging of solid-state NMR observables [19]. Because no other aromatic residues at positions 2 and 22, one helical turn away from the Trp side chains at positions 5 and 19, have yet been addressed, we have investigated the dynamics of peptide helices with histidine residues, which are both aromatic and positively charged (at low pH), in these two positions.

The experiments and analysis indicate that the H^2,22^WALP peptide adopts a well-defined tilt in lipid bilayers and expresses only moderate levels of dynamic averaging (σρ). Its tilt (τ_0_) also scales with the bilayer thickness, from 26° in DLPC to 6° in DOPC. The rotational preferences (ρ_0_) also remain similar to those of the host peptide G^2,22^WALP23. This means that H2 and H22, when flanking a transmembrane helix, confer similar properties to those displayed previously by non-aromatic residues such as glycine, lysine or arginine [13]. Notably, the tryptophan and histidine side chains (H2, W5 and W19, H22) are positioned approximately one helical turn apart and are near the membrane interface. 

Now these types of interactions between histidine and tryptophan side chains are not unusual. A relevant example of such an interaction is found in the M2 proton channel of Influenza-A virus, where the transmembrane segment of the M2 channel contains a HxxxW motif. Indeed the interaction between His37 and Trp41 is essential for the proton selective activity of M2 channels [39,40]. The protonated form of the imidazole ring of His37 of one helix interacts with the indole ring of Trp41 to facilitate the channel gating [39,40,41,42]. The presence of the same sequence motif in the Influenza-B M2 channel (His19 and Trp23) [43,44] strengthens the importance of this type of interaction. Along with the HxxxW motif, the B/M2 channel has an additional histidine residue His27 [45], which suggests an interaction also between Trp23 and His27. Indeed, it is found that the proton conductance decreases to about 60% for a mutation of His27 to Ala [46]. 

Our results with derivatives of GWALP23 support the stabilizing effect of such interactions. Rather than competing for favorable interactions with the polar lipid head groups as observed for the multiple Trp side chains in W^2,22^WALP, the histidine side chains of residues H2 and H22 are able to find proper orientations and to complement Trp interactions with lipid head groups. 

Removal of H22 from the sequence of H^2,22^WALP23 (to give G22) does not affect the helix dynamics or orientation much. The peptide helix continues to display well-defined tilted transmembrane orientations in DLPC and DOPC membranes, which do not change over a pH range of 2–8 in DOPC. In DLPC, there are some changes in the spectral resolution which agree with similar observations for H^2,22^WALP and therefore may provide additional support for the deprotonation of histidine H2 at pH above 4. The key variables defining the dynamic properties remain similar, especially the “rotational slippage” (σρ) of the helix, which remains below 50° in both lipid bilayers. 

In the case of H^22^GWALP23, when H22 is introduced in the absence of H2, in DLPC and DOPC lipids, the helix dynamic behavior changes significantly. The σρ value for rotational slippage increases from 8° in DLPC to a large 70° in DOPC membranes (Table 3). Interestingly, this type of dynamic averaging is diminished when a second histidine is added to position 2 (producing H^2,22^WALP). As mentioned in the Results section, the rotational slippage (σρ) of H^2,22^WALP in both DLPC and DOPC is somewhat in between the respective values observed for H^2^GWALP and H^22^GWALP. The results indicate that H2 and H22 affect largely the motional averaging of the helix, while the actual preferred mean orientations are still controlled by tryptophans W5 and W19. Overall, these helices display well-behaved moderately dynamic tilted transmembrane orientations. 

Even though histidine is a polar and potentially charged residue, its ionization behavior depends on the polarity of its immediate environment. With two –NH groups and a pKa of around 6–7, the imidazole ring can be charged as well as neutral depending on changes in pH and the local dielectric. As such, the pKa can be modulated by the local membrane environment and can drop by 2 to 4 units when the imidazole side chain is buried within the hydrophobic core of a lipid bilayer [20]. For the surface-accessible histidines, nevertheless, there are no observable change in the ^2^H NMR spectra from pH 2 to 8 for any of the labeled alanines in the core helices that are flanked by the H2, H5, H19, H22, or the paired H2,22 or H5,19 substitutions. While the lack of core helix response “could” imply that the histidine side chains prefer only one ionization state, either charged or neutral, such a scenario would seem unlikely. A more likely possibility would suggest that the interfacially located imidazole rings of histidine titrate normally, but the peptide helix does not respond with any change in its preferred orientation or dynamics from pH 2–8. If the helix does not respond to a change in His ionization, then the titration will not be detected by the ^2^H NMR spectra from the core alanines.

In this study, we have characterized the peptides H^5^GWALP23, H^19^GWALP23, GH^5,19^ALP23, and H^2^GWALP23, H^22^GWALP23, H^2,22^WALP23, each containing one or a pair of interfacial histidine residues in DOPC or DLPC lipid membranes. It was previously seen that introduction of two adjacent His residues near the N-terminal, in the case of H^4,5^GWALP23, increases the rotational slippage (σρ) around the helix axis [34]. Here we observe that removal of H4 reduces σρ, while maintaining a well-defined helix tilt angle and azimuthal rotation. The present experiments also show that a change from W5 to H5 causes a shift of -30° in ρ_o_, while the change from W19 to H19 causes an opposite shift of about +20° in ρ_o_, compared to the parent GWALP23 helix with W5 and W19 as a control. Overall, the rotational preferences of the H5W19 and W5H19 peptides differ by ∼50°. Notably, residues 5 and 19 project 40° apart on a helical wheel. With both H5 and H19 present in the same helix, the rotational “corrections” cancel, and the rotational preference once again matches that for GWALP23, when W5 and W19 are present. Moreover, these results projecting the trend of helix rotation are consistent with the trends when tyrosines replace the tryptophan residues [28]. The differences in helix tilt and rotation with respect to anchor group identity (H or W or Y) and location (position 5 or 19) remain similar. A key distinction is the helical unwinding, involving alanine 7 of the core helix, that is displayed in the presence of histidine H5, but not with Trp or Tyr in the same position. Indeed, this particular helix unraveling occurs also for H^4,5^GWALP23 [34]. Such behavior is absent when the histidine is moved to the C-terminal end (in the case of H^19^GWALP23). 

## 5. Conclusions

The present study indicates that single or multiple histidine residues at a membrane interface do not significantly perturb the orientation or dynamics of a transmembrane α-helix. Apparently, the His ionization or otherwise general identity of aromatic rings flanking a core helix seems not to affect the helix dynamics as long as there are not “too many” aromatic rings [19]. Histidine H5 nevertheless uniquely extends the N-terminal unraveling up to residue 7. Fraying of the helix ends has been deemed a crucial factor for defining a particular favored helix orientation and limiting the dynamics [34,35]. The detailed properties of transmembrane helices depend upon the precise locations and interactions of interfacial aromatic residues. 

## Figures and Tables

**Table 1:** Sequences of GWALP23 peptides with single and double histidine substitutions.

Name of peptide	Sequence ^a^	Reference
GWALP23	acetyl-G**G**AL**W**LALALALALALAL**W**LA**G**A-amide	[12]
H^2,22^WALP23	acetyl-G**H**AL**W**LALALALALALAL**W**LA**H**A-amide	This work
H^2^GWALP23	acetyl-G**H**AL**W**LALALALALALAL**W**LA**G**A-amide	This work
H^22^GWALP23	acetyl-GGAL**W**LALALALALALAL**W**LA**H**A-amide	This work
H^5^GWALP23	acetyl-GGAL**H**LALALALALALAL**W**LAGA-amide	This work
H^19^GWALP23	acetyl-GGAL**W**LALALALALALAL**H**LAGA-amide	This work
GH^5,19^ALP23	acetyl-GGAL**H**LALALALALALAL**H**LAGA-amide	This work

^a^ Positions of modifications with histidine (**H**) are shown as **bold** letters and underlined.

**Table 2:** ^2^H-NMR quadrupolar splitting magnitudes of labeled core alanines of GWALP23 family peptides with single and double histidine substitutions.

Lipid	Peptide	[d4] Ala CD3 Quadrupolar Splittings in kHz^a^					
		7	9	11	13	15	17
DLPC	GWALP23	26.4	25.5	26.9	14.6	20.7	3.4
	H^2,22^	30.8	35	29.3	19.4	24.7	4.2
	H^2^	31.9	35.6	31.4	23.7	25.8	1.2
	H^22^	26.3	24.3	25.4	11.7	19.0	7.2
DOPC	GWALP23	16.6	1.7	16.7	1.5	15.4	2.6
	H^2,22^	15.4	1.2	17.0	1.6	16.0	2.1
	H^2^	19.1	6.3	19.5	5.2	17.4	1.6
	H^22^	14.0	1.4	14.1	1.1	12.5	4.1
	H^5,19^	22.3	7.3	18.6	3.8	17.5	1.1
	H^5^	22.1	6.0	16.8	1.2	11.7	8.5
	H^19^	17.3	4.6	19.8	8.2	20.5	5.4

^a^ Values listed are for β = 0^0^ sample orientations. Spectra for the histidine-containing peptides were recorded at pH 4. Values for GWALP23 (unbuffered; pH about 6) are from [13].

**Table 3:** Semistatic GALA and Modified Gaussian analysis results for transmembrane helix orientations of histidine-containing peptides of the GWALP23 family.

Peptide	Lipid	GALA Analysis				Modified Gaussian Analysis^a^				
		τ	ρ	S_zz_	RMSD (kHz)	τ_o_	ρ_o_	στ	σρ	RMSD (kHz)
GWALP^b^	DLPC	20.7°	307°	0.71	0.66	23°	304°	15°	33°	0.7
H2,22		26°	304°	0.74	1.05	24°	303°	10°	16°	1.43
H2		26°	308°	0.77	0.86	33°	305°	10°	36°	0.61
H22		22.7°	297°	0.67	0.66	17°	299°	10°	8°	1.22
GWALP^b^	DOPC	6°	323°	0.87	0.61	9°	321°	9°	48°	0.7
H2,22		6°	329°	0.86	0.33	10°	326°	10°	56°	0.56
H2		8.7°	319°	0.83	0.52	12°	318°	10°	46°	0.52
H22		6°	315°	0.73	0.35	9°	319°	10°	70°	0.45
^c^H5,19		8.7°	319°	0.80	0.97	10°	319°	10°	34°	1.1
^c^H5		8.7°	292°	0.76	0.37	9°	290°	10°	32°	0.6
H19		9.7°	343°	0.83	0.97	11°	342°	10°	28°	0.84

^a^The modified Gaussian analysis followed Sparks et al [19], with S_zz_ fixed at 0.88 and στ fixed at the value shown in the table; ^b^Results for GWALP23 (denoted “GWALP”) are from [13] and [19]; ^c^Calculations were performed without the A7 data point.

## Supplementary Materials

The following are available online at https://www.mdpi.com/2218-273X/10/2/273/s1, Figure S1: MALDI mass spectra of synthesized H^2,22^WALP23, H^2^GWALP23 and H^22^GWALP23, Figure S2: MALDI mass spectra of synthesized GH^5,19^ALP; H^5^GWALP and H^19^GWALP, Figure S3: Circular Dichroism spectra of H^2,22^WALP23; H^2^GWALP23, and H^22^GWALP23 helices in DLPC lipid vesicles, Figure S4: Circular Dichroism spectra of H^5^GWALP23, H^19^GWALP23 and GH^5,19^ALP23 in DOPC lipid vesicles, Figure S5: ^31^P NMR spectra of H^2,22^WALP23, H^2^GWALP23 and H^22^GWALP23 in DLPC lipid bilayers Figure S6: 31P NMR spectra of GWALP23 like peptides with tryptophan to histidine replacements in DOPC lipid bilayers, Figure S7: ^2^H NMR spectra of H^2,22^WALP23, H^2^GWALP23, H^22^GWALP23 in DLPC lipid bilayer, Figure S8: ^2^H NMR spectra of labeled core alanines of H^5^GWALP23, H^19^GWALP23 and GH^5,19^ALP23 in DOPC lipid bilayers, Figure S9: Selected ^2^H NMR spectra to show the pH dependence of resonances for labeled A7 and A9 of H^5^GWALP23 in DOPC lipid bilayers, Figure S10: Selected ^2^H NMR spectra to show the pH dependence of resonances for labeled A7 and A9 of H^19^GWALP23 in DOPC lipid bilayers.
