# Supplementary material for: Comparing Interfacial Trp, Interfacial His and pH Dependence for the Anchoring of Tilted Transmembrane Helical Peptides
Source: Biomolecules. 2020 Feb 11;10(2):273. doi: 10.3390/biom10020273 (PMC7072424; doi:10.3390/biom10020273)
Supplement: Supplementary file 1 [file biomolecules-10-00273-s001.pdf]

Comparing Interfacial Trp, Interfacial His and pH Dependence for the Anchoring of Tilted Transmembrane Helical Peptides

Fahmida Afrose and Roger E. Koeppe II

Department of Chemistry and Biochemistry, University of Arkansas,
Fayetteville, AR, United States 72701

Supplementary Materials

Figure S1: MALDI mass spectra of synthesized $H^{2,22}$WALP23, H^{2}GWALP23 and H^{22}GWALP23.

Figure S2: MALDI mass spectra of synthesized $GH^{5,19}$ALP23; H^{5}GWALP23 and H^{19}GWALP23

Figure S3: Circular Dichroism spectra of $H^{2,22}$WALP23; H^{2}GWALP23, and H^{22}GWALP23 helices in DLPC lipid vesicles.

Figure S4: Circular Dichroism spectra of H^{5}GWALP23, H^{19}GWALP23 and $GH^{5,19}$ALP23 in DOPC lipid vesicles.

Figure S5: ^{31}P NMR spectra of samples with $H^{2,22}$WALP23, H^{2}GWALP23 and H^{22}GWALP23 in DLPC lipid bilayers.

Figure S6: ^{31}P NMR spectra of samples with GWALP23 like peptides with tryptophan to histidine replacements in DOPC lipid bilayers.

Figure S7: ^{2}H NMR spectra of $H^{2,22}$WALP23, H^{2}GWALP23, H^{22}GWALP23 in DLPC lipid bilayers.

Figure S8: ^{2}H NMR spectra of labeled core alanines of H^{5}GWALP23, H^{19}GWALP23 and $GH^{5,19}$ALP23 in DOPC lipid bilayers.

Figure S9: Selected ^{2}H NMR spectra to show the pH dependence of resonances for labeled A7 and A9 of H^{5}GWALP23 in DOPC lipid bilayers.

Figure S10: Selected ^{2}H NMR spectra to show the pH dependence of resonances for labeled A7 and A9 of H^{19}GWALP23 in DOPC lipid bilayers.

Figure S1.

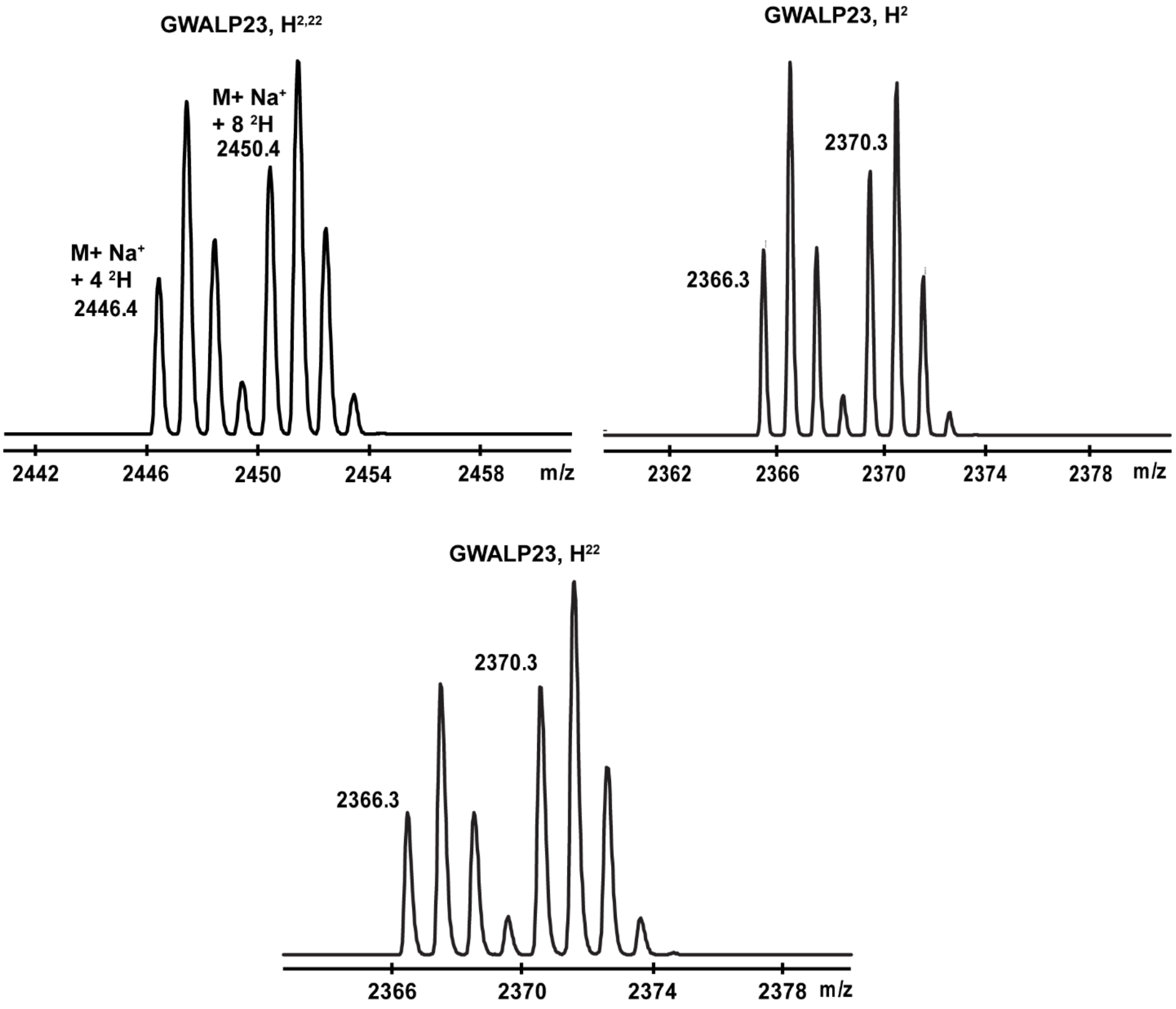

Figure S1: MALDI mass spectra of synthesized $H^{2,22}$WALP23, H^{2}GWALP23 and H^{22}GWALP23.

Figure S2.

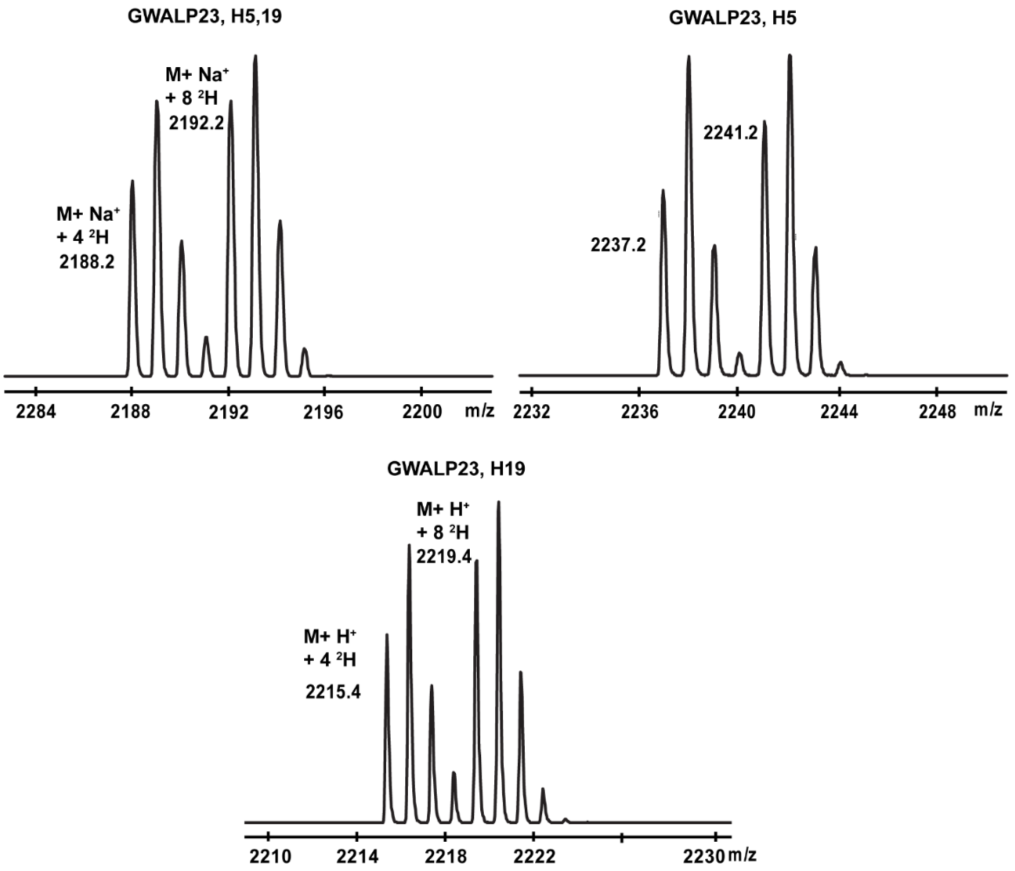

Figure S2: MALDI mass spectra of synthesized GH^{5,19}ALP23; H^{5}GWALP23 and H^{19}GWALP23

Figure S3.

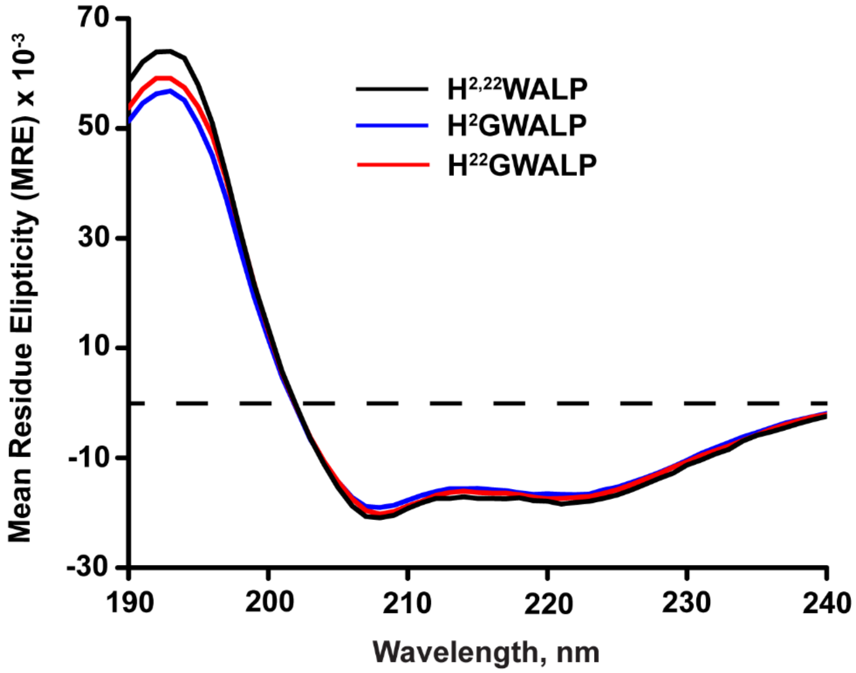

Figure S3: Circular Dichroism spectra of $H^{2,22}$WALP23; H^2GWALP23, and H^{22}GWALP23 helices in DLPC lipid vesicles.

Figure S4.

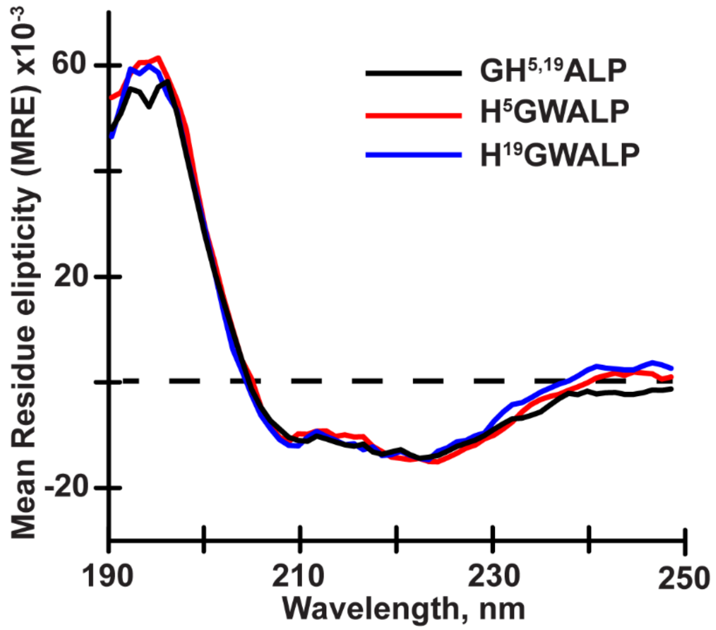

Figure S4: Circular Dichroism spectra of H^{5}GWALP23, H^{19}GWALP23 and GH^{5,19}ALP23 in DOPC lipid vesicles.

Figure S5.

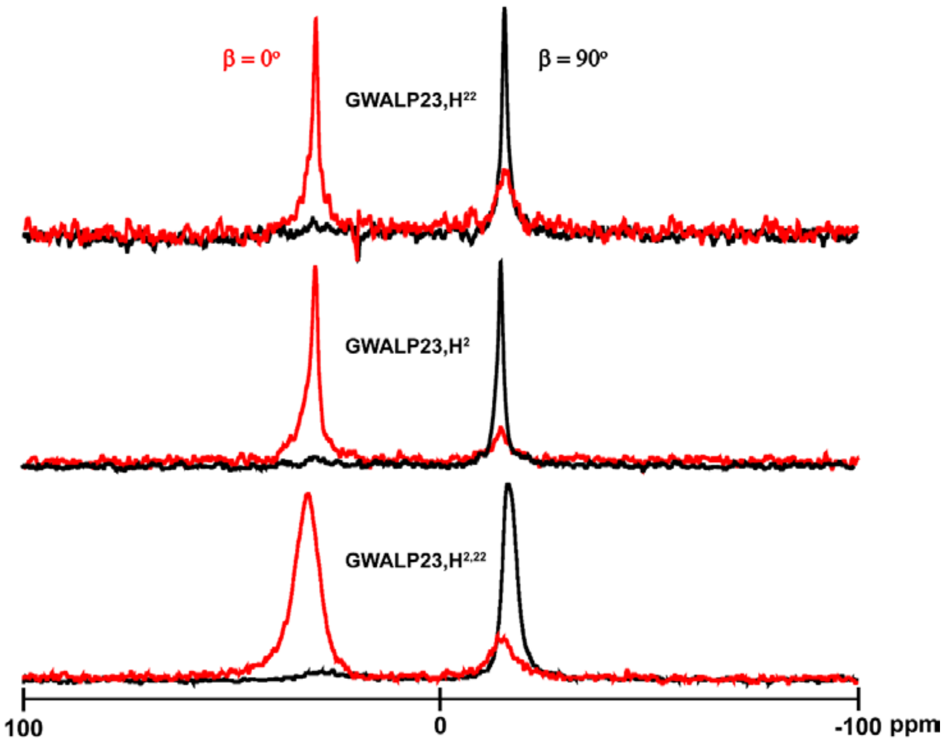

Figure S5: ^{31}P NMR spectra of samples with $H^{2,22}$WALP23, H^{2}GWALP23 and H^{22}GWALP23 in DLPC lipid bilayers.

Figure S6.

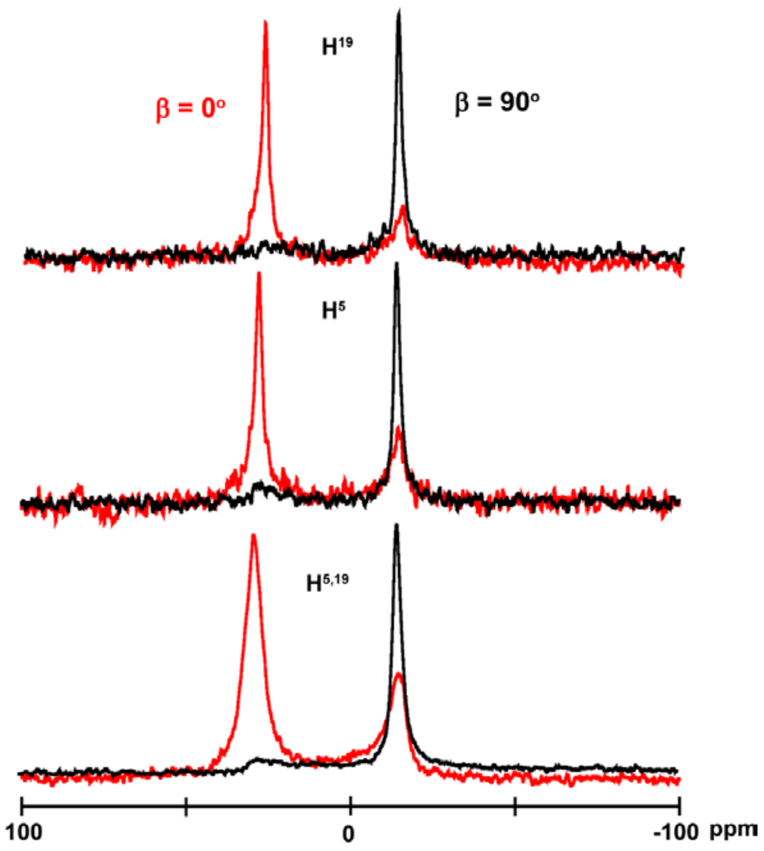

Figure S6: ^{31}P NMR spectra of samples with GWALP23 like peptides with tryptophan to histidine replacements in DOPC lipid bilayers.

Figure S7.

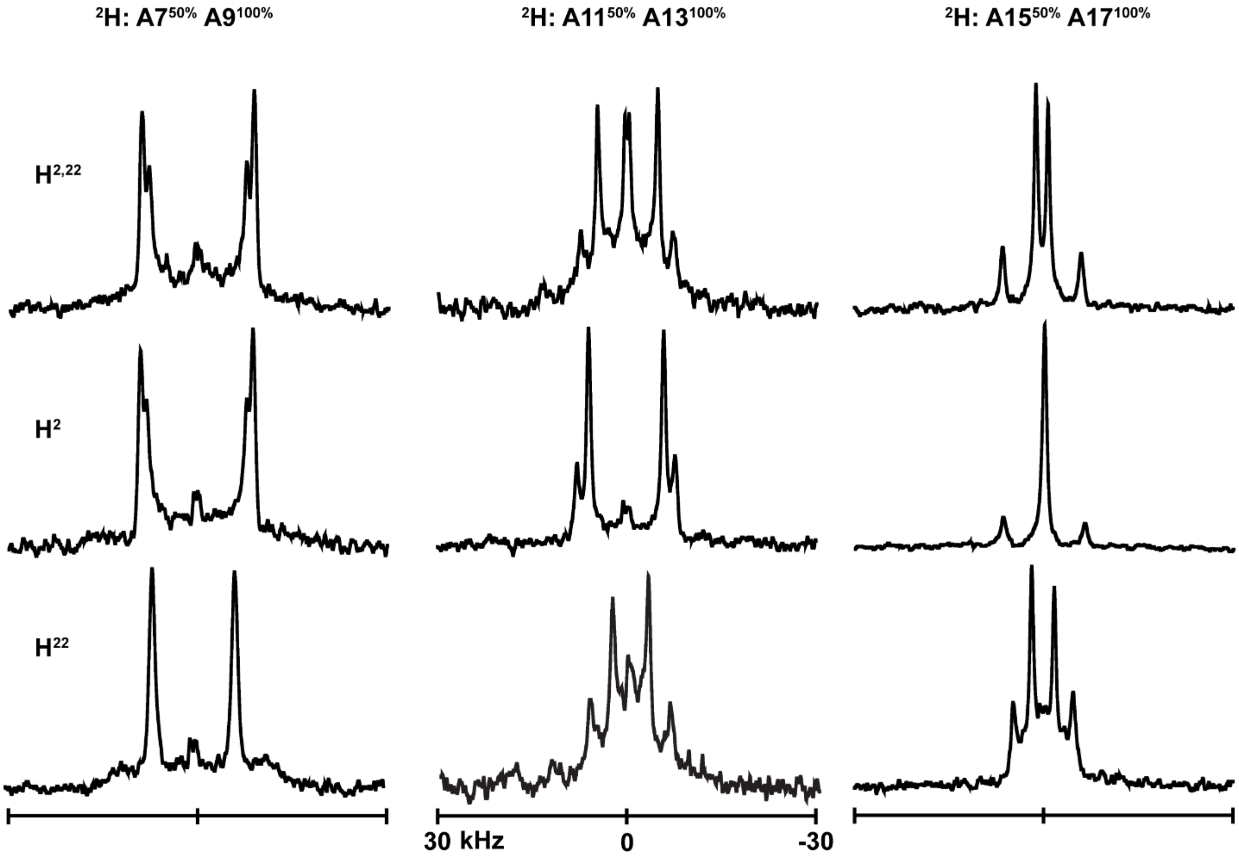

Figure S7: ^{2}H NMR spectra of H^{2,22}WALP23, H^{2}GWALP23, H^{22}GWALP23 in DLPC lipid bilayers.

Figure S8.

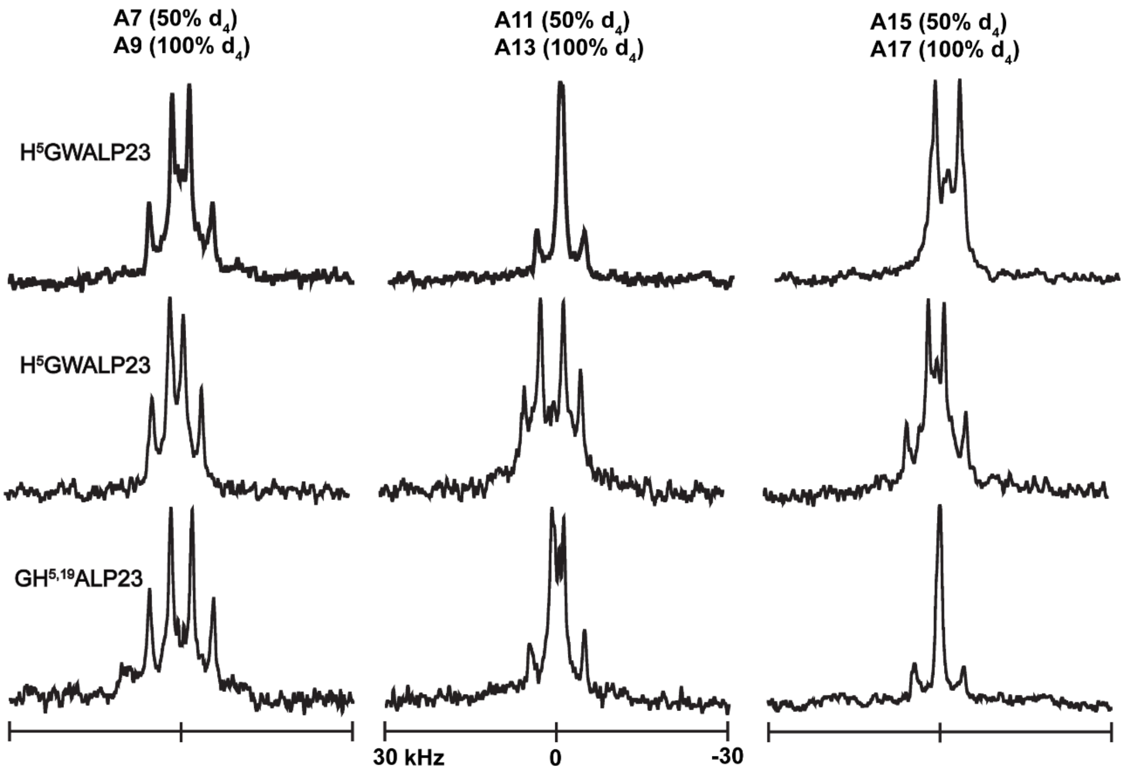

Figure S8: ^{2}H NMR spectra of labeled core alanines of $H^{5}GWALP23$, $H^{19}GWALP23$ and $GH^{5,19}ALP23$ in DOPC lipid bilayers.

Figure S9.

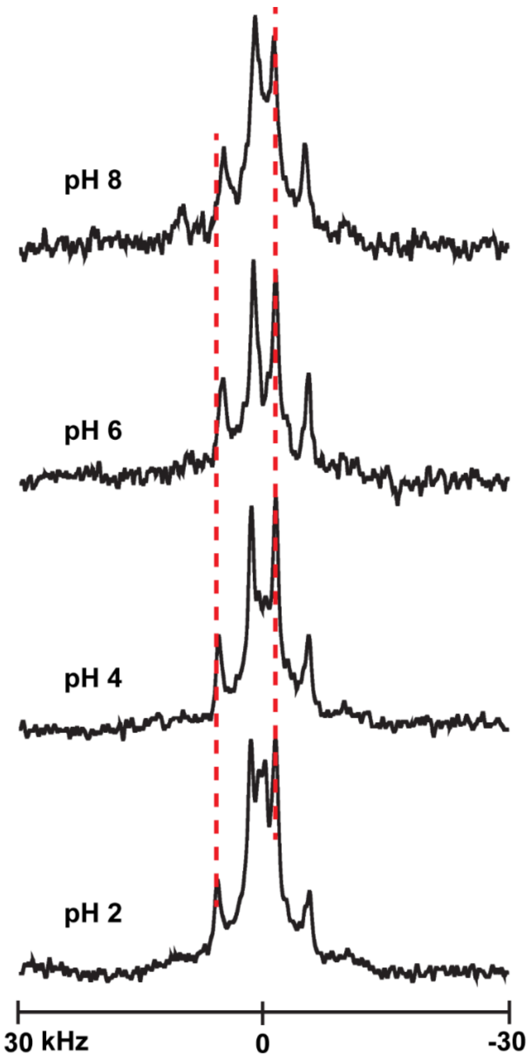

Figure S9: Selected ^{2}H NMR spectra to show the pH dependence of resonances for labeled A7 and A9 of H^{5}GWALP23 in DOPC lipid bilayers.

Figure S10.

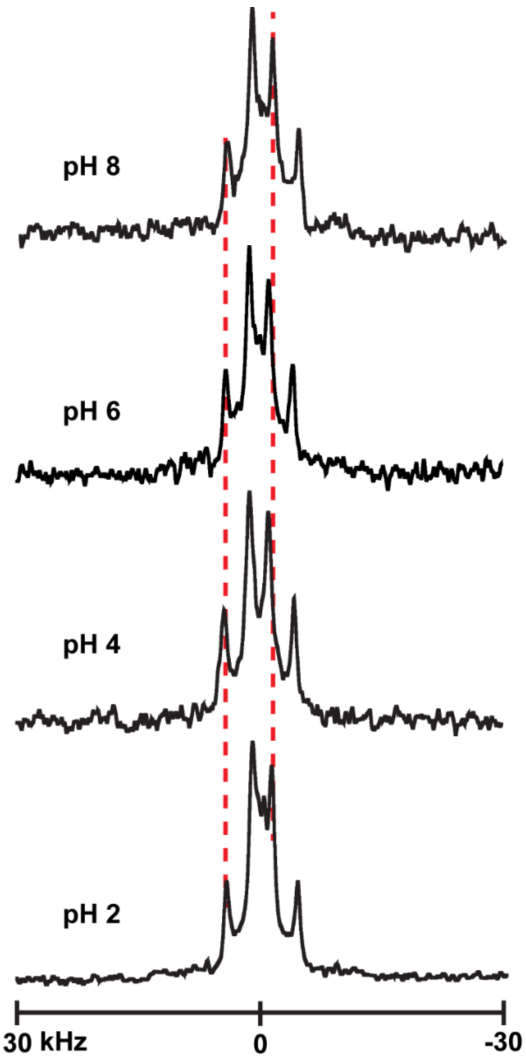

Figure S10: Selected ^{2}H NMR spectra to show the pH dependence of resonances for labeled A7 and A9 of H^{19}GWALP23 in DOPC lipid bilayers.
